# Nitro-fatty acids decrease type I interferons and monocyte chemoattractant protein 1 in ex vivo models of inflammatory arthritis

**DOI:** 10.1186/s12865-021-00471-3

**Published:** 2021-12-17

**Authors:** A. L. Hansen, L. S. J. Rahbek, A. S. Sørensen, M. P. Hundahl, S. Lomholt, C. K. Holm, Tue W. Kragstrup

**Affiliations:** 1 Department of Biomedicine, Aarhus University, Høegh-Guldbergs Gade 10, C. F. Møllers Allé 6, 8000 Aarhus C, Denmark; 2 Department of Rheumatology, Aarhus University Hospital, Aarhus, Denmark; 3 Department of Rheumatology, Silkeborg Regional Hospital, Silkeborg, Denmark

**Keywords:** Nitro-fatty acid, Immunosuppressive drug, Antirheumatic drug, Inflammation, Autoimmunity, Arthritis

## Abstract

**Background:**

Inflammatory arthritis including rheumatoid arthritis (RA) and spondyloarthritis (SpA) is characterized by inflammation and destruction of the joints. Approximately one third of patients do not respond to first-line treatments. Nitro-fatty acids are bioactive lipids with anti-inflammatory properties and tissue-protective functions. The nitro-fatty acid 10-NO_2_-oleic acid (10-NO_2_-OA) is being tested in clinical trials for patients with fibrotic and inflammatory conditions. Here, we tested whether 10-NO_2_-OA could inhibit immune reactions involved in the inflammatory and joint destructive processes in inflammatory arthritis.

**Methods:**

Synovial fluid and blood samples were obtained from 14 patients with active RA or SpA. The in vitro models consisted of synovial fluid mononuclear cells (SFMCs) cultured for 48 h, SFMCs cultured for 21 days, and fibroblast-like synovial cells (FLSs) co-cultured with peripheral blood mononuclear cells (PBMCs) for 48 h. Cells were treated with or without 10-NO_2_-OA or the tumor necrosis factor alpha (TNFα) inhibitor etanercept. Supernatants were analyzed for type I interferon, monocyte chemoattractant protein-1 (MCP-1), matrix metalloproteinase 3 (MMP3) and tartrate resistant acid phosphatase (TRAP).

**Results:**

In SFMCs cultured for 48 h, 10-NO_2_-OA dose-dependently decreased the secretion of bioactive type I interferons and MCP-1 but not MMP3 (*P* = 0.032, *P* = 0.0001, and *P* = 0.58, respectively). Both MCP-1 and MMP3 were decreased by etanercept (*P* = 0.0031 and *P* = 0.026, respectively). In SFMCs cultured for 21 days, 10-NO_2_-OA significantly decreased the production of MCP-1 but not TRAP (*P* = 0.027 and *P* = 0.1523, respectively). Etanercept decreased the production of TRAP but not MCP-1 (*P* < 0.001 and *P* = 0.84, respectively). In co-cultures of FLSs and PBMCs, 10-NO_2_-OA decreased the production of MCP-1 (*P* < 0.0001). This decrease in MCP-1 production was not seen with etanercept treatment (*P* = 0.47).

**Conclusion:**

10-NO_2_-OA decreased the release of MCP-1 in three models of inflammatory arthritis. Of particular interest, 10-NO_2_-OA inhibited type I interferon, and 10-NO_2_-OA was more effective in reducing MCP-1 production in cultures dominated by FLSs compared with etanercept. Our results encourage clinical investigations of 10-NO_2_-OA in patients with inflammatory arthritis.

**Supplementary Information:**

The online version contains supplementary material available at 10.1186/s12865-021-00471-3.

## Introduction

Rheumatoid arthritis (RA), psoriatic arthritis, and spondyloarthritis (SpA) belong to the group of inflammatory arthritis, which is a group of diseases characterized by synovitis and cartilage and bone destruction. Early immune suppression with disease modifying antirheumatic drugs (DMARDs) has drastically improved the management of inflammatory arthritis [1]. However, approximately one third of patients do not respond to first-line treatments, and some patients have inadequate responses to several different drugs [2]. Further, current treatments only dampen dsease activity and do not lead to cure of disease. For this reason, there is an ongoing search for new ways to suppress pathogenic mechanisms in these diseases.

Approximately 20 years ago, a new group of bioactive lipids with anti-inflammatory properties and tissue-protective functions was discovered [3]. These nitro-fatty acids are formed upon a non-enzymatic nitration by nitrogen dioxide (NO_2_) of unsaturated fatty acids [4] (such as conjugated linoleic acid [5] and oleic acids). The nitro-fatty acids are formed endogenously under basal metabolic conditions in humans and levels are increased during various inflammatory conditions [6–8] and infection [9]. Nitro-fatty acids can posttranslationally modify target proteins through Michael addition reactions resulting in *S*-nitro-alkylations at available cysteines [10]. This leads to anti-inflammatory and antioxidative changes via modulation of downstream signaling events in pathways such as nuclear factor-κB (NFκB) [10], peroxisome proliferator-activated receptor γ (PPARγ) [11, 12], and nuclear factor erythroid 2-related factor 2/Kelch- like erythroid cell-derived protein with CNC homology-associated protein 1 (Nrf2/Keap1) [13]. 10-nitro oleic acid (10-NO_2_-OA) is now being studied in fibrotic and inflammatory conditions. The drug has been shown to be safe and well-tolerated in a phase 1 study [14] and is being investigated in glomerulosclerosis (NCT03422510) and kidney injury (NCT02248051).

Recently, nitro-fatty acids have been shown to inhibit the production of type I interferons via stimulator of interferon genes (STING) [9]. STING is essential for the production of type I interferon in the host response to infections with DNA viruses [15–17] and bacteria [18–21]. STING requires posttranslational palmitoylation at two conserved cysteine residues (cysteine 88 and cysteine 91) for its activation, signaling and subsequent type I interferon response [22]. Nitro-fatty acids have been shown to nitroalkylate the thiol group of exactly these two cysteine residues in STING. This inhibits the downstream STING-signaling which in turn inhibits the production of interferons [9, 23].

Autocrine type I interferon signalling in dendritic cells can cause an inflammatory arthritis phenotype in mice [24] and the interferon signature is increased in early arthritis [25]. Therefore, targeting the production of interferons might serve as a potential treatment of inflammatory arthritis. A variety of other cytokines, chemokines and enzymes are involved in the arthritogenic processes of inflammatory arthritis [26]. The chemokine monocyte chemoattractant protein-1 (MCP-1) is increased and acts as a significant mediator of inflammation and macrophage infiltration in the joint [27, 28]. Matrix metalloproteinase 3 (MMP3) can cleave the proteoglycan part of articular cartilage [29] and tartrate resistant acid phosphatase (TRAP) is produced by osteoclasts resulting in bone resorption [30].

Here, we investigate the effect of 10-NO_2_-OA on the production of type I interferons, MCP-1, MMP3, and TRAP in ex vivo models mimicking the different components of the disease processes in inflammatory arthritis. 10-NO_2_-OA showed an inhibitory effect on type I interferon production. In all three models of inflammatory arthritis, 10-NO_2_-OA treatment resulted in a decrease in MCP-1 production. There was no effect of 10-NO_2_-OA on MMP3 or TRAP secretion.

## Materials and methods

### Patients and samples

A cross-sectional, paired set of synovial fluid mononuclear cells (SFMCs) and peripheral blood mononuclear cells (PBMCs) were obtained from patients with chronic RA (n = 8) or peripheral SpA (n = 6) with at least one swollen joint (from where synovial fluid was acquired) at the outpatient clinic at Aarhus University Hospital at the time of therapeutic arthrocentesis as previously described (Table 1) [31–33]. Not all patient samples were used in all experiments. The exact number of patients in each experiment has been stated in the figure legends and shown in Additional file 1: Fig. S1.

**Table 1:** Patient characteristics

Age (years)	37 (29–60)
Gender (female)	8
Diagnosis	
Rheumatoid arthritis	8
Peripheral spondyloarthritis	6
Disease duration (years)	6.5 (1.5–19)
Treatment	
Methotrexate	6
Sulfasalazine	3
TNF inhibitor	3
Disease activity	
CRP (mg/L)	20.0 (6.5–55.0)
DAS28CRP (0–10)	3.56 (3.1–4.5)

Data are expressed as median (interquartile range). Information on disease duration, treatment, and disease activity was missing in 4 patients. TNF; tumor necrosis factor; C-reactive protein; DAS28CRP, Disease activity score based on CRP and number of tender and swollen joints in 28 joints

### Culture conditions and read out

The nitrated oleic acid 10-NO_2_-OA (Cayman Chemicals) was used at 5 μM and 10 μM diluted in ethanol (same concentratiosn as used previously) [9]. In all experiments, cells cultured with the TNFα inhibitor etanercept at 5 μg/ml was used as a treatment control (same concentratiosn as used previously) [33]. Untreated cells or cells treated with ethanol (vehicle) were used as matched negative controls. Supernatants were harvested after centrifugation of the culture plates at 1200 rpm for 5 min and the cell-free supernatant was thereafter stored at − 80 °C for later analysis. Results with the etanercept treatment control has partially been published previously together with another dataset [33]. An MTT assay was used to measure cell viability (Roche) (Additional file 1: Fig. S2).

### SFMC 48-h ex vivo model

The 48-h culture was used as an in vitro model of inflammatory arthritis dominated by lymphocytes and monocytes. SFMCs were isolated and cultured as described previously (n = 12) [31]. Briefly, SFMCs were isolated by Ficoll-Paque (GE Healthcare) density-gradient centrifugation and frozen at − 135 °C. The cells were then thawed and seeded at a concentration of 1 × 10^6^ cells/ml in Dulbecco’s modified Eagle’s medium with 10% fetal calf serum, penicillin, streptomycin, and glutamine (culture medium) for 48 h and kept in a humidified incubator at 37 °C and 5% CO_2_. After 48 h, the cell-free culture supernatants were analyzed for the concentration of type I interferons, MCP-1, and MMP3 (Additional file 1: Fig. S3).

### SFMC 21-day ex vivo model

The 21-day culture was used as a model of low-grade inflammatory osteoclastogenesis [34]. The cultures were performed as described for the 48-h model, changing the medium and renewal of incubation condition every 2–3 days for a total of 21 days (n = 9) [31]. This means that cultures were either untreated, or treated with ethanol control, 10-NO_2_-OA 5 μM or 10 μM, or etanercept for the entire period. Culture supernatants were analyzed for the concentration of MCP-1 and the enzyme activity of tartrate-resistant acid phosphatase (TRAP) (Additional file 1: Fig. S3).

### FLS-PBMC 48-h ex vivo model

FLSs were grown from SFMCs and used for analyses at passage 4–5 as previously described [35]. The FLS-PBMC co-culture was used as an in vitro model of inflammatory arthritis modelling the interactions between FLSs and mononuclear cells migrating to the inflamed joint. Briefly, FLSs were grown from SFMCs changing the culture medium every 2–3 days. When the cell layer was 70% confluent after approximately 14 days, the FLSs were passaged by trypsin/EDTA treatment and used for analyses at passage 4–5. To start the co-culture, the FLSs were cultured with paired autologous PBMCs in culture medium for 48 h (n = 6). Culture supernatants were analyzed for the concentration of MCP-1 and MMP3 (Additional file 1 Fig. S4).

### MCP-1 and MMP3 ELISA and TRAP measurement

The concentration of MCP-1 (Biolegend, San Diego, CA, USA) and MMP3 (R&D Systems) was analyzed by commercially available enzyme-linked immunosorbent assays (ELISA) according to the manufacturer’s instructions. TRAP was analyzed by an enzymatic assay (B-bridge International, CA, USA) according to the manufacturer’s instructions as done previously [34].

### HEK-Blue interferon-α/β Cell assay

Human type I interferon bioactivity was analyzed by utilizing the commercially available reporter cell line HEK-Blue interferon-α/β (InvivoGen) according to the manufacturer’s instructions. SEAP levels were determined using a spectrophometer at OD 620 nm.

### Statistics

Statistical analyses and graphs were done using GraphPad Prism 9 for Mac (GraphPad Software, La Jolla, CA, USA). All data were transformed to ratios by dividing the value of the samples with the value of either untreated cultures (etanercept) or ethanol treated cultures (10-NO2-OA). Ratios were then log transformed and analyzed with repeated measures one-way ANOVA (testing for dose–response relationship) or the paired t-test (comparing treatment group with negative control group). A two-sided *P* value < 0.05 was considered statistically significant.

## Results

### 10-NO_2_-OA decreases bioactive type I interferon production by SFMCs cultured for 48 h

**Fig. 1:**
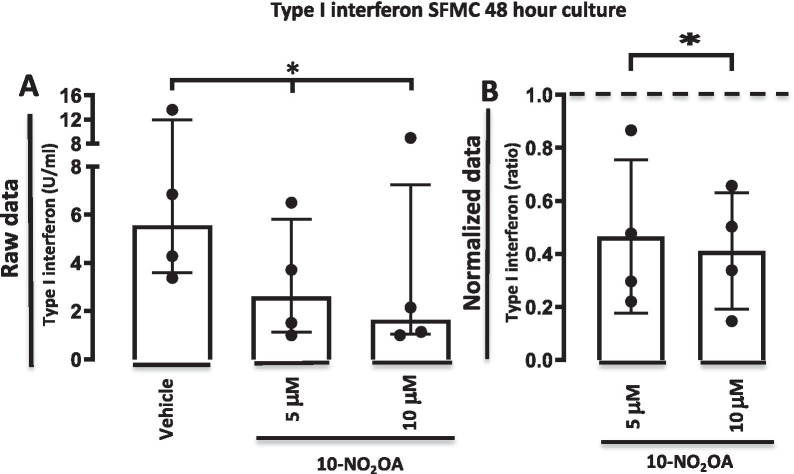
Type I interferon bioactivity in SFMC 48-h cultures incubated with vehicle or 10-NO_2_-OA at 5 μM or 10 μM. **A** Data *shown* as type I interferon bioactivity (U/ml). Bars indicate median and interquartile range. **B** Data *shown* as ratios of the type I interferon bioactivity in treated cultures divided by the bioactivity in the control culture. Dotted line represent the type I interferon bioactivity in the control culture. Bars indicate mean and SD. * = *P* < 0.05

Nitro-fatty acids have previously been shown to function via inhibition of type I interferon production. Therefore, the secretion of type I interferons by SFMCs cultured for 48 h was assessed. The 10-NO_2_-OA significantly decreased bioactive type I interferons dose-dependently (*P* = 0.032). The decrease was not significant when separately comparing cultures treated with 10-NO_2_-OA either at 5 μM (*P* = 0.057) or at 10 μM (*P* = 0.052) and the negative control cultures treated with ethanol (Fig. 1).

### 10-NO_2_-OA reduces MCP-1 but not MMP3 production by SFMCs cultured for 48 h

**Fig. 2:**
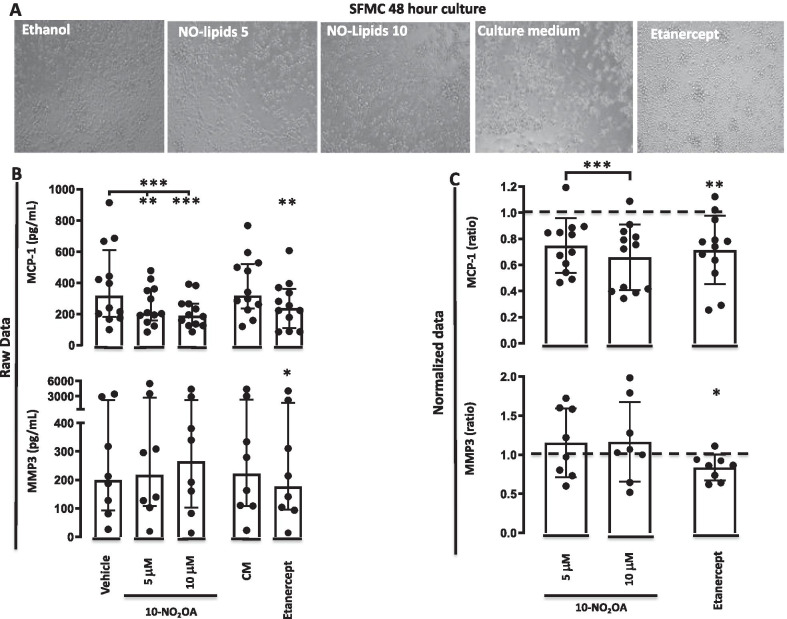
MCP-1 and MMP3 concentration in SFMC 48-h cultures incubated with vehicle or 10-NO_2_-OA at 5 μM or 10 μM, culture medium or etanercept. **A** Light microscope cell culture representative images. **B** Data shown as concentrations (pg/ml). Bars indicate median and interquartile range. **C** Data shown as ratios of the MCP-1 or MMP3 concentration in treated cultures divided by the concentration in the control cultures. Dotted line represent the ratio of the control cultures. Bars indicate mean and SD. * = *P* < 0.05, *** = *P* < 0.001. CM; culture medium

We then tested whether 10-NO_2_-OA affects the secretion of the proinflammatory chemokine MCP-1 by SFMCs. The 10-NO_2_-OA significantly decreased the production of MCP-1 both at 5 μM (*P* = 0.0015), at 10 μM (*P* = 0.0006) and in a dose dependent manner (*P* = 0.0001). Etanercept also significantly decreased the production of MCP-1 (*P* = 0.0031) (Fig. 2). We then tested whether 10-NO_2_-OA affects the secretion of the connective tissue matrix degrading enzyme MMP3 by SFMCs. The 10-NO_2_-OA did not alter the production of MMP3 at neither the 5 μM concentration (*P* = 0.36), the 10 μM concentration (*P* = 0.39) nor dose-dependently (*P* = 0.58). In contrast, etanercept significantly decreased the production of MMP3 (*P* = 0.026) (Fig. 2).

### 10-NO_2_-OA reduces MCP-1 production but not inflammatory osteoclastogenesis in SFMCs cultured for 21 days

**Fig. 3:**
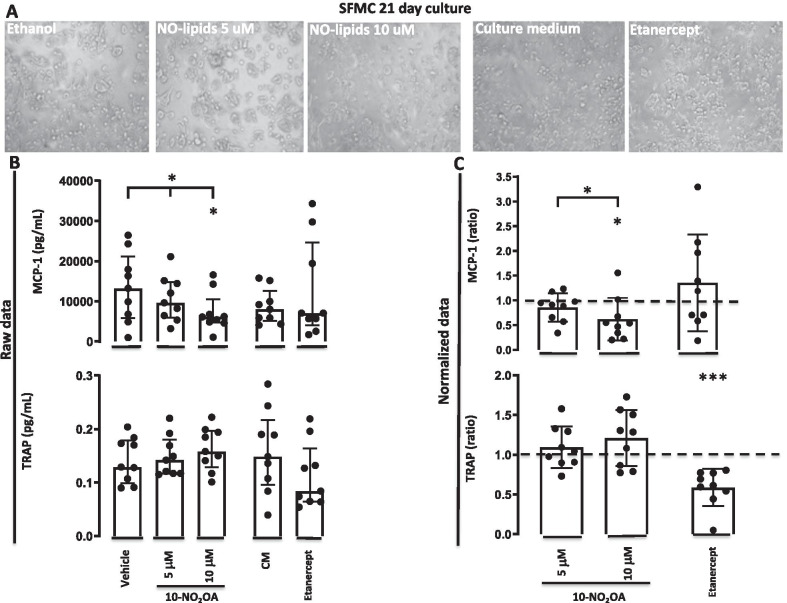
MCP-1 concentration and TRAP activity in SFMC 21-day cultures incubated with vehicle or 10-NO_2_-OA at 5 μM or 10 μM, culture medium or etanercept. **A** Light microscope cell culture representative images. **B** Data shown as concentration (pg/ml) or enzyme activity (OD). Bars indicate median and interquartile range. **C** Data shown as ratios of the MCP-1 concentration or TRAP activity in treated cultures divided by the concentration or enzyme activity in the control cultures. Dotted line represent the ratio of the control cultures. Bars indicate mean and SD. * = *P* < 0.05, *** = *P* < 0.001. CM; culture medium

Then, the effect of 10-NO_2_-OA on the secretion of TRAP and MCP-1 by SFMCs cultured for 21 days was studied to evaluate the immune modulatory capacity of 10-NO_2_-OA in this model of inflammatory osteoclastogenesis. The 10-NO_2_-OA did not alter the production of TRAP at neither the 5 μM concentration (*P* = 0.31), the 10 μM concentration (*P* = 0.11) nor dose-dependently (*P* = 0.15). Etanercept significantly reduced the production of TRAP (*P* = 0.0001) (Fig. 3). The 10-NO_2_-OA significantly decreased the production of MCP-1 at 10 μM (*P* = 0.029) and in a dose dependent manner (*P* = 0.027). There was no statistically significant reduction in cultures treated with 10-NO_2_-OA at 5 μM (*P* = 0.17). There was no difference between cultures treated with etanercept and the negative untreated control cultures (*P* = 0.84) (Fig. 3).

### 10-NO_2_-OA reduces MCP-1 production but not MMP3 production by co-cultures of FLSs and autologous PBMCs

**Fig. 4:**
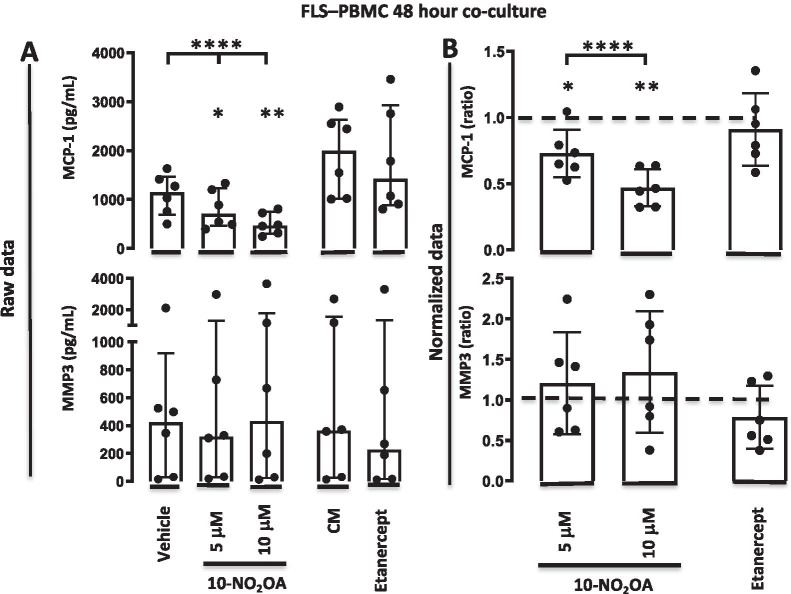
MCP-1 and MMP3 concentrations in FLS-PBMC 48-h co-cultures incubated with vehicle or 10-NO_2_-OA at 5 μM or 10 μM, culture medium or etanercept. **A** Data shown as concentrations (pg/ml). Bars indicate median and interquartile range. **B** Data shown as ratios of the MCP-1 or MMP3 concentration in treated cultures divided by the concentration in the control cultures. Dotted line represent the ratio of the control cultures. Bars indicate mean and SD. **** = *P* < 0.0001. CM; culture medium

Next, the effect of 10-NO_2_-OA was evaluated in the co-cultures of FLSs and autologous PBMCs. The 10-NO_2_-OA significantly decreased the production of MCP-1 both at 5 μM (*P* = 0.014), at 10 μM (*P* = 0.0089) and in a dose dependent manner (*P* < 0.0001). This decrease in MCP-1 production was not seen with etanercept treatment (*P* = 0.47). Neither 10-NO_2_-OA (*P* = 0.31) nor etanercept (*P* = 0.24) decreased the production of MMP3 by the co-cultures of FLSs and autologous PBMCs (Fig. 4).

## Discussion

Approximately one third of patients with inflammatory arthritis do not benefit from treatment with current available first-line DMARDs. Therefore, there is a need for target validation and further drug development to improve therapeutic management of these patients. Nitro-fatty acids modify inflammatory pathways and 10-NO_2_-OA is now being studied as a therapeutic drug in glomerulosclerosis and kidney injury. In this study, we tested whether 10-NO_2_-OA decreases the production of type I interferons, MCP-1, MMP3, and TRAP in ex vivo models mimicking the different components of the disease process in inflammatory arthritis.

Recently, nitro-fatty acids were shown to decrease type I interferon production by directly targeting STING activation and signaling [9]. Therefore, we first tested whether 10-NO_2_-OA could alter the production of type I interferons in SFMCs from inflammatory arthritis patients. In the 48-h culture, 10-NO_2_-OA significantly reduced the production of type I interferons. Interferons are known to play a crucial role in the immune host defense against viral infections [36]. In RA, the interferon signature is increased in early disease [25] and has been associated with poor response to B cell depleting therapy with rituximab [37]. Therefore, 10-NO_2_-OA could be a novel option in RA patients with a high interferon signature.

Hereafter, we compared the effects of 10-NO_2_-OA with the effects of the TNFα inhibitor etanercept across three ex vivo models of inflammatory arthritis. In SFMCs cultured for 48-h, 10-NO_2_-OA significantly reduced MCP-1 production in a dose dependent manner. The 48-h SFMC culture primarily consists of lymphocytes and monocytes. 10-NO_2_-OA did not significantly reduce MMP3 production. Etanercept decreased both the production of MCP-1 and MMP3. This indicates that 10-NO_2_-OA might not prevent the structural damage mediated by MMP3. However, it is not known whether other mediaters of cartilage degradation could be affected by 10-NO_2_-OA.

The 21-day culture resembles inflammatory osteoclastogenesis. 10-NO_2_-OA had no effect on the production of TRAP-1 in this model while etanercept decreased TRAP production. This indicates that 10-NO_2_-OA might not prevent osteoclastogenesis in inflammatory arthritis. However, it is not known whether 10-NO_2_-OA could influence other aspects of osteoclast activation and bone destruction. Notably, 10-NO_2_-OA decreased MCP-1 production while etanercept had no effect on the production of MCP-1 in this model.

The FLS-PBMC co-cultures simulate the interactions of the synovial FLS with monocytes and lymphocytes. 10-NO_2_-OA reduced the production of MCP-1 in the co-culture but not the production of MMP3. Etanercept showed no effect on either MCP-1 or MMP3 levels. This points to a possible role of using 10-NO_2_-OA in FLS-dominated inflammatory conditions.

Overall, there were several differences between 10-NO_2_-OA and etanercept. This is interesting because it underlines that the mechanism of action is very different for the two compounds. 10-NO_2_-OA modulates downstream signaling via NFκB, PPARγ, Nrf2/Keap1, and STING. Etanercept binds and neutralizes the proinflammatory cytokine TNFα. Therefore, 10-NO_2_-OA could be a valueable addition in the treatment of inflammatory arthritis. Inflammatory arthritis are heterogeneous diseases. E.g., based on histological findings in synovial biopsies RA can be divided in the three pathotypes (l) lympho-myeloid, (2) diffuse-myeloid, and (3) pauci-immune characterised by prevalent stromal cells [38]. Interestingly, the pathotype dominated by FLS seemingly responds worse to treatment than other pathotypes [39, 40]. Therefore, treatment options for this subset of patients is especially needed. Further investigations of 10-NO_2_-OA in specifically difficult to treat RA could therefore be interesting.

There are several limitations to this study. First, we only measured type I interferons, MCP-1, MMP3, and TRAP. Many other cytokines, chemokines and enzymes are relevant in the pathogenesis of inflammatory arthritis. Further, SFMCs are rather heterogeneous with large interdonor variations. The proportion of lymphocytes and monocytes varies greatly and other cell types such as fibroblasts, endothelial cells and dendritic cells could also present. Therefore, the findings in this study are not necessarily generalizable.

**Fig. 5:**
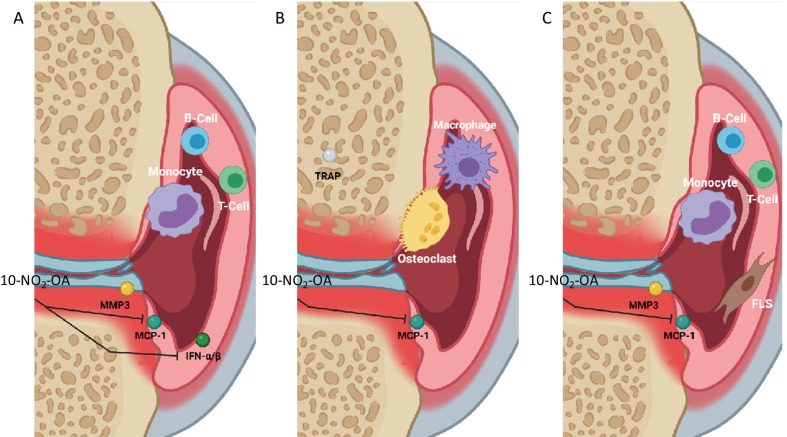
Graphical summary of the results. **A** In SFMCs cultured for 48 h, 10-NO_2_-OA decreased the secretion of bioactive type I interferons and MCP-1 but not MMP3. **B** In SFMCs cultured for 21 days, 10-NO_2_-OA decreased the production of MCP-1 but not TRAP. **C** In co-cultures of FLSs and PBMCs, 10-NO_2_-OA decreased the production of MCP-1 but not MMP3

To summarize, 10-NO_2_-OA decreases MCP-1 in three models of inflammatory arthritis (Table 2 and Fig. 5). 10-NO_2_-OA seems to be a novel mode of action for suppressing pathogenic mechanisms in inflammatory arthritis. Interestingly, 10-NO_2_-OA inhibited type I interferon production and 10-NO_2_-OA was more effective in reducing MCP-1 production in cultures dominated by FLSs compared with etanercept. Therefore, our results encourage clinical investigations of 10-NO_2_-OA in patients with inflammatory arthritis. This treatment would be a new option especially in inflammatory arthritis patients with a high interferon signature or with a fibroblast dominated pathotype.

**Table 2:**
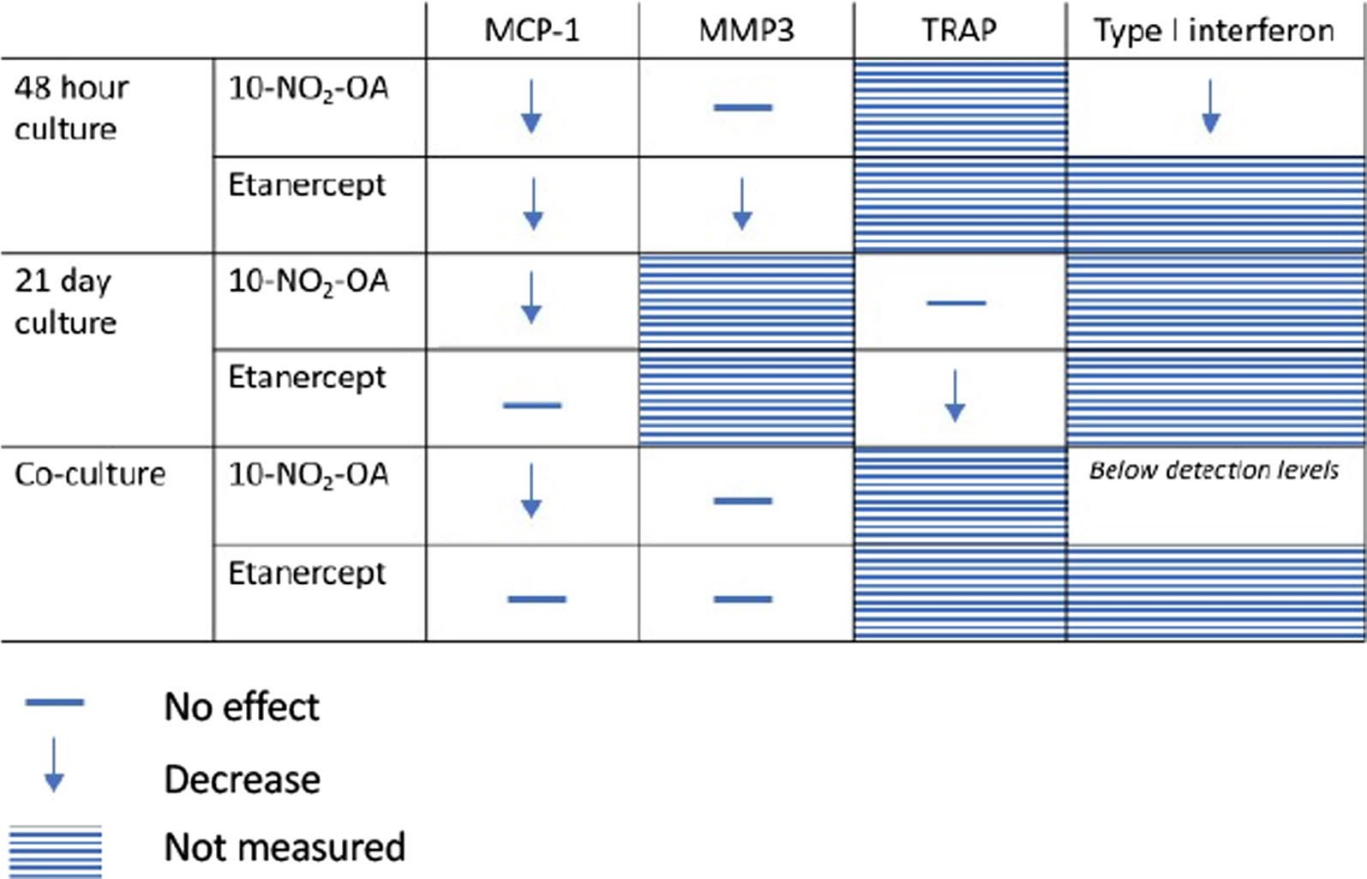
Summary of results

In SFMCs cultured for 48 h, 10-NO_2_-OA decreased the secretion of bioactive type I interferons and MCP-1 but not MMP3. In SFMCs cultured for 21 days, 10-NO_2_-OA decreased the production of MCP-1 but not TRAP. In co-cultures of FLSs and PBMCs, 10-NO_2_-OA decreased the production of MCP-1 but not MMP3

## Supplementary Information

**Additional file 1. Figure S1**. Flowchart showing the samples used in each experiment.** Figure S2**. MTT assay with synovial fluid mononuclear cells and fibroblast-like synovial cells.** Figure S3**. Overview of experimental setup for SFMC 48-hour and SFMC 21-day culture models.** Figure S4**. Overview of experimental setup for the FLS-PBMC co-culture model.

## Data Availability

Please contact corresponding author for data requests (email: kragstrup@biomed.au.dk).

## Abbreviations

DMARD: Disease-modifying antirheumatic drug
ELISA: Enzyme-linked immunosorbent assay
FLS: Fibroblast-like synovial cell
MCP-1: Monocyte chemoattractant protein-1
MMP3: Matrix metalloproteinase 3
PBMC: Peripheral blood mononuclear cell
RA: Rheumatoid arthritis
SFMC: Synovial fluid mononuclear cell
SpA: Spondyloarthritis
TNF: Tumor necrosis factor
TRAP: Tartrate-resistant acid phosphatase
