# Supplementary material for: Nitro-fatty acids decrease type I interferons and monocyte chemoattractant protein 1 in ex vivo models of inflammatory arthritis
Source: BMC Immunol. 2021 Dec 17;22:77. doi: 10.1186/s12865-021-00471-3 (PMC8684285; doi:10.1186/s12865-021-00471-3)
Supplement: Supplementary file 1 — Additional file 1. Figure S1. Flowchart showing the samples used in each experiment. Figure S2. MTT assay with synovial fluid mononuclear cells and fibroblast-like synovial cells. Figure S3. Overview of experimental setup for SFMC 48-hour and SFMC 21-day culture models. Figure S4. Overview of experimental setup for the FLS-PBMC co-culture model. [file 12865_2021_471_MOESM1_ESM.docx]

# Aditional file 1. Figures S1-4.


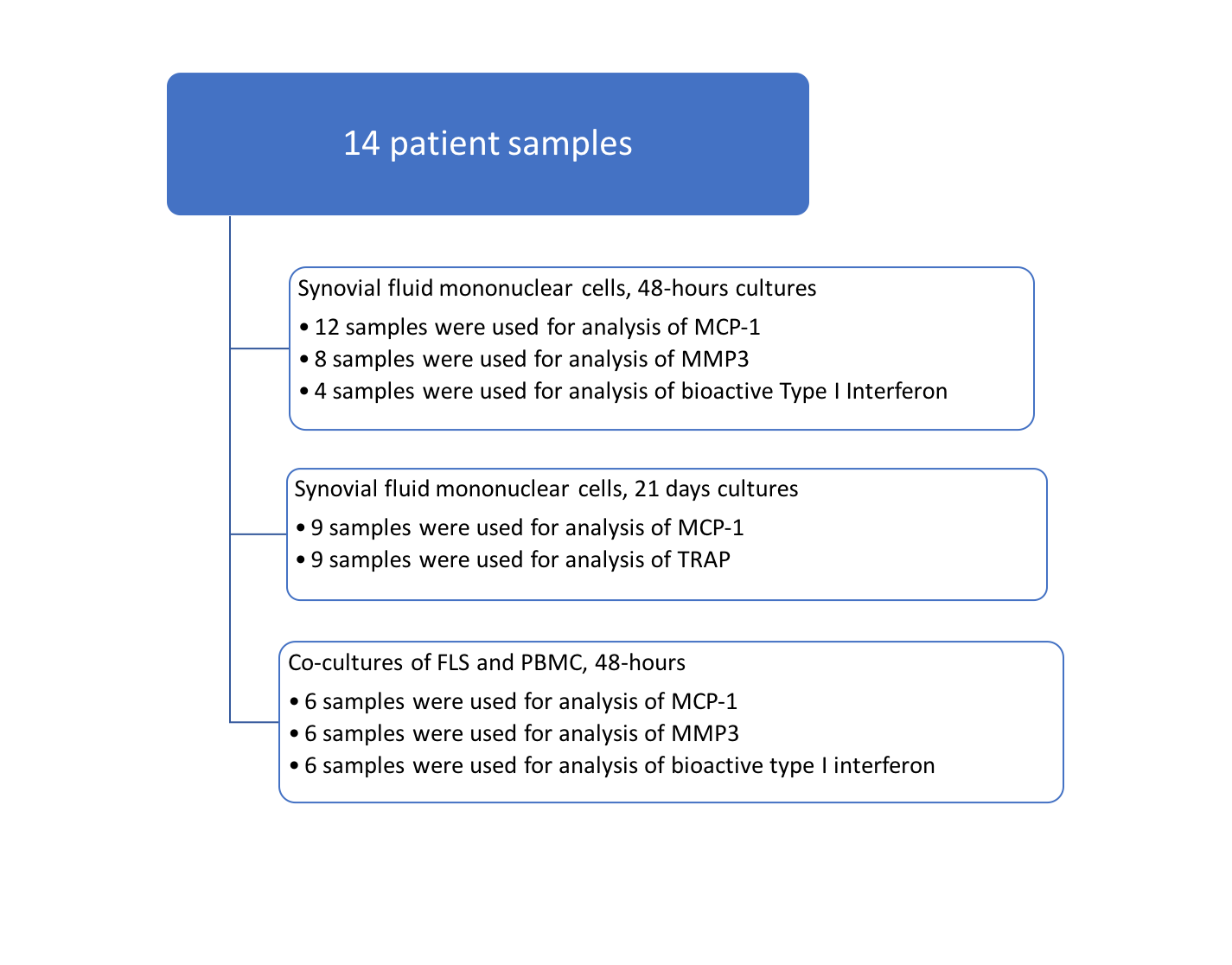


Figure S1. Flowchart showing the samples used in each experiment.

Figure S2. MTT assay with synovial fluid mononuclear cells and fibroblast-like synovial cells.


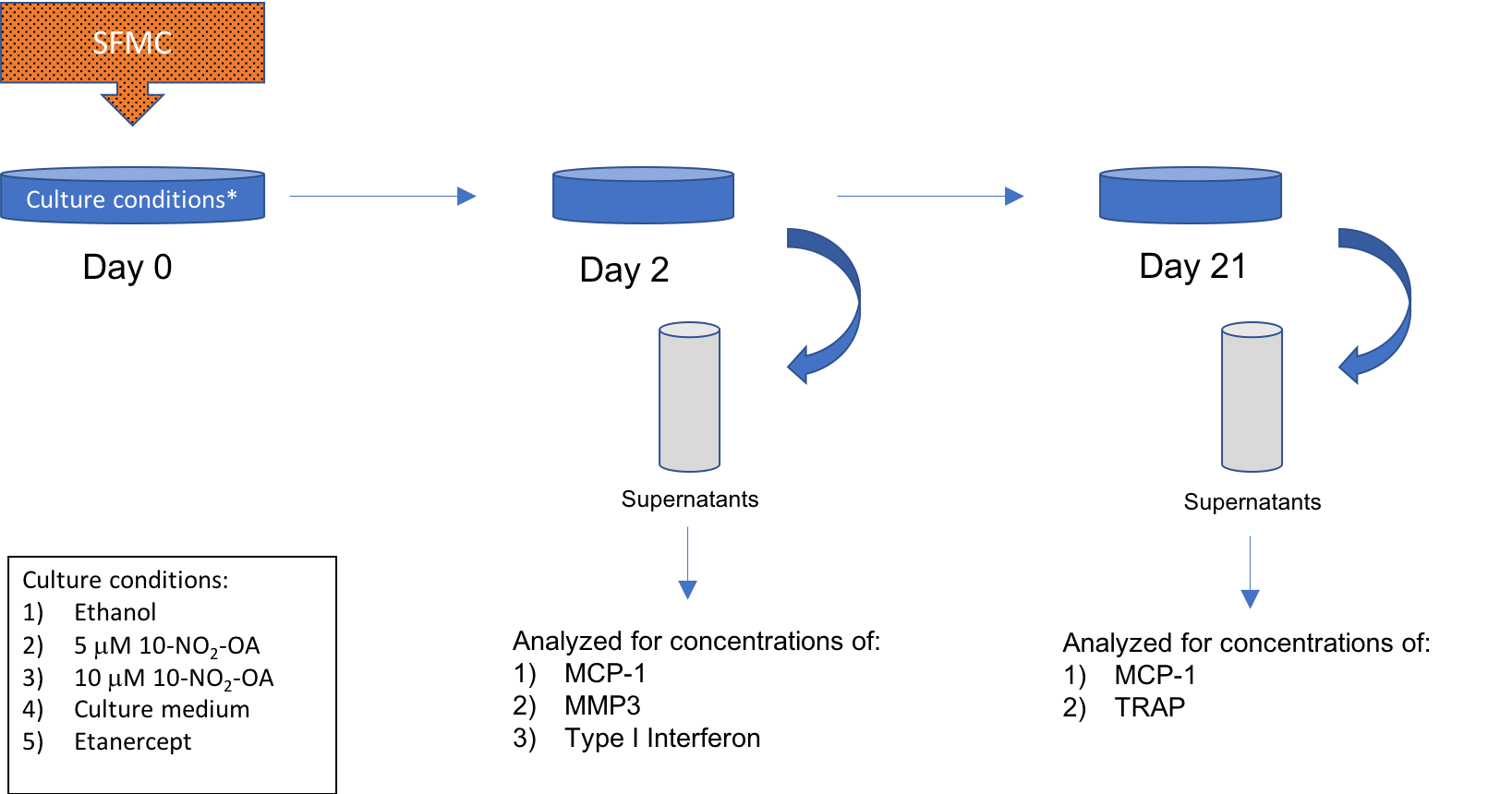


**Figure S3**. Overview of experimental setup for SFMC 48-hour and SFMC 21-day culture models.


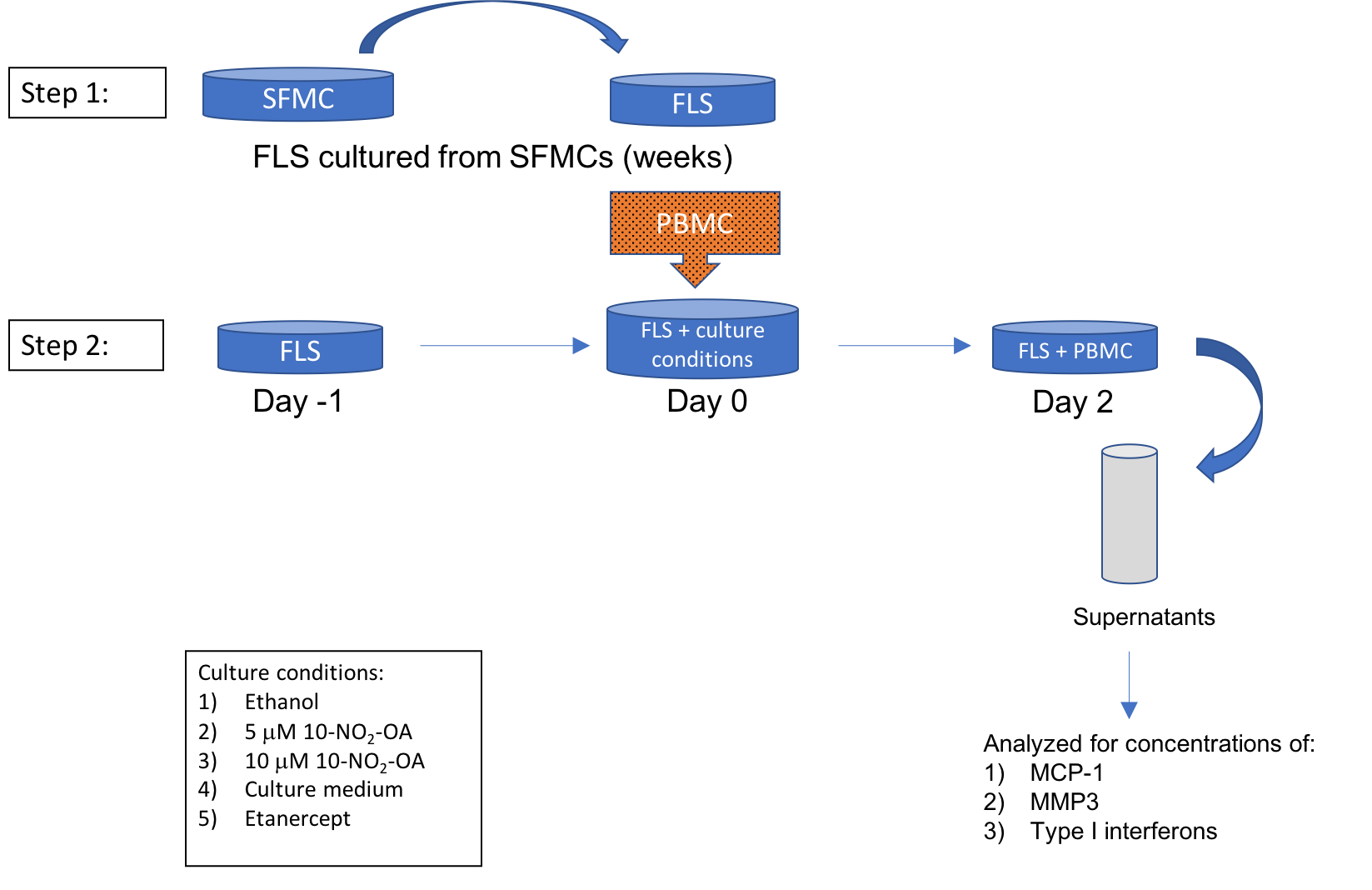


**Figure S4**: Overview of experimental setup for the FLS-PBMC co-culture model.
